# Unraveling the Convoluted Biological Roles of Type I Interferons in Infection and Immunity: A Way Forward for Therapeutics and Vaccine Design

**DOI:** 10.3389/fimmu.2014.00412

**Published:** 2014-08-29

**Authors:** Danushka Kumara Wijesundara, Yang Xi, Charani Ranasinghe

**Affiliations:** ^1^Virology Laboratory, Department of Surgery, Basil Hetzel Institute, University of Adelaide, Adelaide, SA, Australia; ^2^Molecular Mucosal Vaccine Immunology Group, The John Curtin School of Medical Research, The Australian National University, Canberra, ACT, Australia; ^3^Lung and Allergy Research Centre, Translational Research Institute, UQ School of Medicine, The University of Queensland, Woolloongabba, QLD, Australia

**Keywords:** type I interferons, human immunodeficiency virus, IFN-ε, vaccine adjuvants, interferon immunity

## Abstract

It has been well-established that type I interferons (IFN-Is) have pleiotropic effects and play an early central role in the control of many acute viral infections. However, their pleiotropic effects are not always beneficial to the host and in fact several reports suggest that the induction of IFN-Is exacerbate disease outcomes against some bacterial and chronic viral infections. In this brief review, we probe into this mystery and try to develop answers based on past and recent studies evaluating the roles of IFN-Is in infection and immunity as this is vital for developing effective IFN-Is based therapeutics and vaccines. We also discuss the biological roles of an emerging IFN-I, namely IFN-ε, and discuss its potential use as a mucosal therapeutic and/or vaccine adjuvant. Overall, we anticipate the discussions generated in this review will provide new insights for better exploiting the biological functions of IFN-Is in developing efficacious therapeutics and vaccines in the future.

## Introduction

Since the initial discovery of type I interferons (IFN-Is) as anti-viral agents (1), these cytokines have been extensively studied for their anti-microbial and immune regulatory properties. IFN-I family comprises 13 IFN-α subunits, IFN-β, IFN-ω, IFN-ε, IFN-κ, IFN-τ, and IFN-δ (in mice only) (2–8). All IFN-Is signal through the IFN-α receptor (IFN-AR) complex to induce synthesis and secretion of IFN-inducible genes or effector proteins with anti-viral, pro-apoptotic, and ubiquitination-modifying properties (9–11). The signaling pathways that IFN-Is utilize to exert various biological effects have been comprehensively reviewed elsewhere and will not be reviewed here [see Ref. (12)]. Numerous cell types produce IFN-Is (e.g., macrophages, myeloid dendritic cells (DCs), fibroblasts, and epithelial cells), but plasmacytoid DCs (pDCs) appear to be the most prolific producers of IFN-Is (13, 14). The production of these cytokines tends to be beneficial to the host particularly against acute viral infections, but there are considerable evidences to suggest that IFN-Is play detrimental roles in autoimmune diseases (15), bacterial and persistent viral infections. Herein, we review how IFN-Is could play beneficial or detrimental roles in pathogen control predominantly with respect to viral infections and discuss how they could be used as therapeutics and vaccine adjuvants. Furthermore, the importance of considering the emerging IFN-ε in immunity and vaccine development will be discussed.

## The Benefits and Detriments of IFN-Is in the Control of Pathogens

The importance of IFN-Is in protecting hosts against pathogens has been demonstrated in several contexts. Firstly, IFN-AR deficient mice tend to be more susceptible to infection with viruses (particularly acute viral infections) compared to wild-type mice. Some examples include Henipavirus (16), acute Friend virus (17), encephalitic flavivirus (18), lymphocytic choriomeningitis virus (LCMV) Armstrong (19), Hazara virus (20), Dengue virus (21), Respiratory Syncytial Virus (22), and numerous other viral infections (23). Secondly, systemic exhaustion of IFN-Is following a primary viral infection has been shown to increase the host susceptibility to secondary unrelated viral infections in mice (24). Thirdly, therapeutic administration of IFN-Is can reduce viral loads in individuals infected with chronic viruses and promote cancer regression (see below Section “The Use of IFN-Is as Therapeutics and Adjuvants”). Finally, pathogens can attenuate IFN-I responses to promote immune evasion. For instance, human immunodeficiency virus (HIV)-1 can reduce the capacity of IFN producing cells to produce IFN-Is (25–27), induce cytopathic effects on these cells (28–32), and/or block IFN-I mediated intracellular signaling events (33) to help establish a chronic phase infection. Similarly, cancer immune evasion and development could also involve attenuation of IFN-I responses. In agreement with this, Critchley-Thorne et al. (34) have shown that various cancer patients have significantly attenuated expression of interferon stimulate genes in lymphocytes compared to healthy controls.

The benefits of IFN-Is in conferring protection against microbes have been mostly demonstrated using acute viral infection models, but several studies suggest that IFN-Is can also assist in the control of bacterial infections. This was first demonstrated *in vitro* where De la Maza and colleagues (35) showed that IFN-I inhibit *Chlamydia trachomatis* infectivity of human and mouse cell lines. Several subsequent studies have shown that IFN-I could indeed play important roles for inhibiting various stages of bacterial infections. Some examples include replication of *Chlamydophila pneumoniae* (36), recruitment of *Myobacterium tuberculosis* target cells into the lung during early infection (37), and invasion and transmigration of *Streptococcus pneumoniae* in the lungs (38). However, IFN-Is do not always appear to render beneficial outcomes in anti-bacterial immunity. Several studies have reported that IFN-AR deficient mice are better protected than WT controls following bacterial infections such as *Ehrlichia muris* (39), *Chlamydia muridarum* (40), *Listeria monocytogenes* (41, 42), *Myobacterium* species (43, 44), and *Francisella tularensis* (45). Furthermore, induction of IFN-Is following virus infections could make hosts more susceptible to secondary bacterial infections (46–48). The mechanisms as to how IFN-Is exacerbate or make hosts more susceptible to bacterial disease may vary depending on the infection. For instance, IFN-I mediated disease exacerbation has been linked to reduction of interleukin (IL)-17 expressing γδ T cells, increased expression of IL-10 or reduction in cell-mediate immune responses following *F. tularensis, M. Leprae*, or *L. monocytogenes*, respectively (42, 44, 45).

Several reports suggest that the detrimental effects of IFN-Is could also support the establishment of persistent viral infections depending on the quantities and duration of IFN-I induction. IFN-Is have been shown to play significant roles in inhibiting various stages (e.g., replication, virus assembly, protein trafficking, and transcription) of HIV-1 life cycle (49–53). However, sustained unlike transient production of IFN-Is resulting from chronic stimulation of pDCs has been proposed to facilitate HIV-1 persistence (54). Similarly following clone 13 LCMV infection transient (within 24 h) hyper-induction of IFN-α and -β has been reported to exacerbate virus pathogenesis and promote viral persistence (19). However, in the same study IFN-Is were crucial for the control of acute Armstrong LCMV infection, which was likely due to lower IFN-I induction following Armstrong compared to clone 13 LCMV infection. In chronic simian immunodeficiency virus (SIV) infection studies, disease free phenotypes of sooty mangabeys have been associated with the abolishment of interferon stimulated gene expression during chronic, but not in acute phase infection (55). Overall, it can be speculated that early, transient yet non-excessive induction of IFN-Is (at least α and β species) are important in the control of acute viral infections. On the contrary, chronic and/or hyper-induction of IFN-Is could provide an environment for enhanced persistence and/or pathogenesis of chronic viral infections.

## IFN-Is and Regulation of Adaptive Immunity

Apart from their most celebrated role as direct anti-viral agents, IFN-Is have also been increasingly recognized as potent regulators of cellular immune responses. Of particular interest to vaccine development has been the ability of these cytokines to regulate adaptive immune responses and this aspect is discussed here.

Dendritic cells are often crucial for initiating adaptive immune responses and serve as important targets for IFN-Is to regulate adaptive immunity. Exposure of IFN-Is facilitates maturation of DCs via increasing the expression of DC-associated chemokine receptors, co-stimulatory molecules, and major histocompatibility complex class I and class II antigen presentation (56–60). Consequently, DCs that mature following IFN-I exposure can effectively prime protective T cell responses (61). A caveat here is that IFN-I responses could operate in a threshold dependent manner where excessive responsiveness is inhibitory to the ability of DCs to prime T cell responses. For instance, following LCMV infection higher induction of IFN-Is has been associated with heightened expression of programed death-ligand 1 (PD-L1) on DCs and PD-L1 interaction with programed death 1 (PD-1) on T cells can inhibit T cell activation (19, 62).

IFN-Is could also act directly on lymphocytes to alter adaptive immune outcomes. Naïve B cells up-regulate the expression of activation markers CD69, CD86, and CD25 following IFN-I exposure *in vitro* (63), but *in vivo* IFN-Is only up-regulate CD69 and CD86 expression on naïve B and T cells (64). The consequences of up-regulating these activation markers are not clear, but *in vitro* studies suggest it could serve to reduce the activation thresholds of naïve B cells unlike T cells (63, 65). Alternatively, CD69 expression resulting from IFN-I exposure can down-regulate sphigosine-1 phosphate receptor-1 on naïve lymphocytes to retain these cells in secondary lymphoid organs (66). This retention mechanism could facilitate a more durable interaction between naïve lymphocytes and DCs for efficient lymphocyte activation to occur. IFN-Is have been reported to represent a distinct third signal for naïve T cell activation to occur and prevent the expansion of regulatory T cells that can inhibit T cell activation (67–69). Furthermore, IFN-Is regulate the functions of lymphocytes even after naïve lymphocyte activation or effector/memory differentiation. Some examples of this include IFN-I mediated enhancement in cell division (63, 70), survival (71, 72), interferon-γ secretion (73), cytotoxicity (74), germinal center formation, and antibody isotype switching (75).

Despite the many studies demonstrating that IFN-Is are capable of boosting adaptive immunity; there have also been several studies in bacterial and chronic viral infection settings suggesting that IFN-I signaling leads to IL-10 production (19, 44, 76, 77). IL-10 is thought to be detrimental to the clearance of these pathogens as has been demonstrated with HIV-1 (78). It is likely that IFN-Is up-regulate PD-1 expression (e.g., on regulatory T cells) and PD-L1 (e.g., on DCs) on cells resulting in a milieu where PD-1/PD-L1 interactions occur; this could facilitate IL-10 production and exhaustion of T cell function during chronic viral infections (19, 76–80). A caveat here is that IFN-Is in some instances can also inhibit IL-10 production and IL-10 production can occur independently of IFN-I signaling (76, 81). Furthermore, IFN-Is up-regulate pro-apoptotic molecules such as Bak on T cells to induce apoptosis independently of T cell exhaustion (82).

Overall, IFN-Is play pivotal roles in boosting adaptive immunity, but the switch from becoming a booster to an inhibitor of adaptive immunity may reflect on how much apoptosis, PD-1/PD-L1 interactions and IL-10 signaling are induced on immune cells due to IFN-Is.

## The Use of IFN-Is as Therapeutics and Adjuvants

The development of efficient methods to purify IFN-I and subsequent high yield purification of IFN-α2 during the late 1970s paved way for the first IFN-I based human clinical trial in 1986 where IFN-α2 was used for treating hairy cell leukemia (83, 84). Since then the therapeutic use of IFN-Is have shown promising outcomes for treatment of several cancers and viral infections. Therapeutic administration of pegylated IFN-α2 have rendered potent anti-viral and immune enhancing effects against hepatitis B virus infection (85, 86). A recent clinical trial has shown that similar outcomes could be achieved even when pegylated IFN-α2 is administered to HIV-infected patients (87). Systemic administration of IFN-α and/or IFN-β has also been reported to reduce viral growth and clinical manifestations of herpes zoster, herpes simplex virus, and cytomegalovirus (CMV) infections (88–91). Furthermore, systemic or intralesional administration of IFN-α and/or IFN-β has been shown to induce a regression of skin-associated wart infections following papilloma virus infections (92–98). IFN-Is have also been used in synergic regimens where administration of IFN-α2 or -β2 and anti-viral drugs (e.g., ribavirin and faldaprevir) could effectively reduce viral loads of certain hepatitis C virus (HCV) genotypes and is currently the best treatment for HCV-infected patients (99–102). A caveat here is that these regimens have also been reported to cause adverse side-effects (103). Apart from treatment of pathogen infections, IFN-Is especially IFN-α2, have also been used for treatment and regression of various cancers (e.g., leukemia, prostrate cancer, and cervical intraepithelial neoplasia) (104–106).

Studies in pre-clinical models suggest that IFN-Is could also be potent vaccine adjuvants for inducing adaptive immune responses. Some examples include when an influenza vaccine adjuvanted with IFN-α/β administered mucosally induced significantly higher IgG2a and IgA antibody responses and protection compared to non-adjuvanted vaccines (107, 108). Interestingly, the species of IFN-Is used as immune adjuvants could have different immune outcomes in terms of enhancing adaptive immunity. Studies in our laboratory suggest that recombinant pox viral vectors encoding IFN-β compared to those encoding IFN-α4 or IFN-ε significantly enhanced systemic T cell immunity against co-encoded antigens in prime-boost vaccination settings (109). However, Xi et al. (110) using similar prime-boost vaccination settings demonstrated that the use of IFN-ε was much more efficient in inducing T cell immunity in mucosal compartments (e.g., lung and gut) compared to IFN-α4 and IFN-β when used as vaccine adjuvants. Another important consideration here is that the vaccine vectors (i.e., pox viruses) used in our studies are acute attenuated viruses and do not chronically induce IFN-Is as is usually the case with persistent virus infections.

There are several confounding factors that could dictate the use of IFN-I in therapy and as vaccine adjuvants. Firstly, unique biological effects have been reported with different members of the IFN-I family and subtypes of IFN-α. Thus, the choice of IFN-I species (e.g., IFN-α2 or IFN-β) could dictate the success of IFN-I treatment or IFN-I based vaccine formulations. Secondly, members of the IFN-I family have different binding affinities and kinetics to the IFN-AR subunits with current comparative studies suggesting that IFN-β has the highest affinity to IFN-AR and anti-viral capacity (111–113). A caveat with these studies is that not all members of the IFN-I family were compared. Thirdly, IFN-Is can cause numerous adverse side-effects and induce autoimmunity (e.g., lupus, thyroiditis, diabetes, dermatitis, Sjogren’s syndrome, and arthritis) especially in patients with a history of autoimmune manifestations (114). The autoimmune outcomes in these settings are thought to be a combination of tolerogenic immune function failures and IFN-I mediated maturation of DCs that present autoantigens to activate autoreactive T cells and B cells that make autoantibodies (115).

Collectively, IFN-Is have shown considerable promise for the treatment of cancers and pathogen infections (e.g., chronic viruses) in some clinical settings. IFN-Is are also promising for use as vaccine adjuvants, but the species of IFN-Is used for this purpose could have a significant bearing on adaptive immunity generated at certain immune compartments. For instance, IFN-β could be used to effectively enhance systemic T cell immune responses, whereas IFN-ε is more promising as an adjuvant to enhance mucosal T cell immunity in the lung and the gut mucosae.

## Importance of IFN-ε in Immunity and Vaccine Development

Most studies investigating the roles of IFN-Is have done so mainly analyzing the roles of IFN-α and -β. However, investigating the roles of other IFN-I family members is beneficial for effective therapeutic and vaccine development strategies especially given that higher induction of IFN-α and -β could be detrimental to the host as discussed previously. For this purpose, it is indeed intriguing to evaluate the roles of IFN-ε, which unlike other IFN-Is is constitutively expressed and plays various protective roles in reproductive tissues, gut, lung, and the brain (Table 1). Since our initial studies characterizing the roles of IFN-ε in inducing anti-viral states on cells (109), we have found that this cytokine also possesses potent immune regulatory capacity. Our recent studies indicated that, intranasal immunization of mice with vaccinia virus (VV) encoding murine IFN-ε (VV-HIV-IFN-ε) unlike IFN-α (VV-HIV-IFN-α4) or IFN-β (VV-HIV-IFN-β) could induce rapid clearance of VV in the lung (110). Viral clearance in this instance correlated with several immune outcomes: (i) elevated lung VV-specific CD8^+^CD107a^+^IFN-γ^+^ cell population expressing activation markers CD69/CD103, (ii) enhanced lymphocyte recruitment to lung alveoli with reduced inflammation, and (iii) highly functional CD8^+^CD4^+^ double positive T cell subset [CD3_high_C–C chemokine receptor (CCR)7_high_CD62L_low_] in lung lymph nodes (110). Next when IFN-ε was used in an intranasal/intramuscular heterologous HIV-1 prime-boost vaccination regimen, elevated HIV-specific effector, but not memory CD8^+^ T cells responses were detected in spleen, genito-rectal nodes, and Peyer’s patches. Furthermore, homing marker α4β7 and CCR9 analysis showed that unlike other IFN-Is, IFN-ε promoted the migration of antigen-specific CD8^+^ T cells to the gut mucosae (110). These results for the first time established that unlike other IFN-Is, IFN-ε played a unique role at the mucosae. Another recent study has also further substantiated our findings demonstrating that IFN-ε deficient mice were more susceptible to intra-vaginal herpes simplex virus 2 and *Chlamydia muridarum* infections compared to wild-type mice (117). This suggests that IFN-ε could also be beneficial for the control of certain bacterial infections. A caveat here is that it is unknown whether IFN-ε could cause adverse side-effects in humans as it has not yet been used for treatment or vaccination purposes in humans.

**Table 1:** **Site-specific effects of IFN-ε**.

Site	Function	Reference
Brain	Maintenance of the structure and function	(116)
Lung	Promote clearance of viral infections	(110)
	Recruitment of unique yet highly anti-viral CD4^+^CD8^+^ T cells	
Gut	Enhance expression of CCR9 and α4β7 on anti-viral T cells to promote homing to the gut (i.e., Peyer’s patches)	(110)
Reproductive tissues	Regulation of embryonic development Protect male and female reproductive tissues against infections (e.g., herpes and *Chlamydia*)	(117, 118)

Overall, IFN-ε has great potential to be used as a topical microbicide or a therapeutic to control local lung/gut infections or modulate tissue-specific immunity at sites where pathogens are initially encountered (i.e., mucosal surfaces). Specifically, IFN-ε’s ability to enhance CD8^+^ T cell homing to the gut [gut is the primary site of HIV virus replication and CD4^+^ T-cell depletion (119)] and also its ability to control infections at the lung mucosae suggest that administration of pegylated forms of IFN-ε or vaccines encoding IFN-ε could be effective for controlling mucosal pathogens such as HIV-1.

## Concluding Remarks

The dual roles of IFN-Is in providing beneficial and detrimental effects to the host in pathogen control is intriguing for developing IFN-I based vaccines and therapies. Lessons learned from acute viral infection models and studies comparing acute versus chronic infection states suggest that transient, but not sustained and/or excessive induction of IFN-Is is likely to confer protective outcomes. IFN-Is have also proven to be promising therapeutic agents against various pathogens and cancers and could also be used as vaccine adjuvants. The caveat here is that the vaccine vector used should ideally not chronically stimulate the production of IFN-Is, which is expected to be detrimental for the generation of robust adaptive immune responses. Our laboratory and others have demonstrated that IFN-ε has great potential to provide protective outcomes against not only mucosal viral infections, but also certain mucosal bacterial infections. Keeping this in mind, more studies need to evaluate the contribution of the different species of IFN-Is not just IFN-α and -β in immunity against infections. These studies are expected to pave way for the development of novel and effective IFN-I based vaccines/therapies against chronic pathogens and cancers.

## Conflict of Interest Statement

The authors declare that the research was conducted in the absence of any commercial or financial relationships that could be construed as a potential conflict of interest.
